# Association of Fully Branded and Standardized e-Cigarette Packaging With Interest in Trying Products Among Youths and Adults in Great Britain

**DOI:** 10.1001/jamanetworkopen.2023.1799

**Published:** 2023-03-14

**Authors:** Eve Taylor, Deborah Arnott, Hazel Cheeseman, David Hammond, Jessica L. Reid, Ann McNeill, Pete Driezen, Katherine East

**Affiliations:** 1 Department of Addictions, King’s College London, London, United Kingdom; 2 Action on Smoking and Health (ASH), London, United Kingdom; 3 Shaping Public Health Policies to Reduce Inequalities and Harm (SPECTRUM) Consortium, Edinburgh, United Kingdom; 4 School of Public Health Sciences, University of Waterloo, Waterloo, Ontario, Canada; 5 Department of Psychology, University of Waterloo, Waterloo, Ontario, Canada

## Abstract

**Importance:**

e-Cigarette vaping among youths and adults has increased in Great Britain. The design of e-cigarette packaging may appeal to youths. Regulations that reduce the appeal of e-cigarettes to youths may deter adult smokers from trying e-cigarettes to help them quit smoking.

**Objective:**

To examine the association of fully branded and standardized e-cigarette packaging with interest in trying products among youths and adults in Great Britain.

**Design, Setting, and Participants:**

In this survey study comprising 2 surveys, the online Action on Smoking and Health Smokefree Great Britain survey collected data between March 25 and April 16, 2021, from a representative sample of 2469 youths (aged 11-18 years) and between February 18 and March 18, 2021, from a representative sample of 12 046 adults (aged ≥18 years).

**Interventions:**

A between-individuals experimental design was used to examine participants’ perceptions of e-cigarette packs that were digitally altered to remove brand imagery and color. Participants were randomly assigned to view a set of 3 e-cigarette packs from 1 of 3 different packaging conditions: (1) fully branded packs (control), (2) white standardized packs with brand name, or (3) green standardized packs with brand name.

**Main Outcomes and Measures:**

Youth participants were asked which product people their age would be most interested in trying, while adult participants were asked which product they would be most interested in trying. All participants could respond “no interest” or “don’t know.” Logistic regression models tested whether reporting no interest in trying the e-cigarettes differed between the pack conditions.

**Results:**

This study included 2469 youths (1286 female youths [52.1%]; mean [SD] age, 15.0 [2.3] years) and 12 046 adults (6412 female [53.2%]; mean [SD] age, 49.9 [17.4] years). Youths had higher odds of reporting no interest among people their age in trying the e-cigarettes packaged in green (292 of 815 [35.8%]; adjusted odds ratio [AOR], 1.37; 95% CI, 1.10-1.71; *P* = .005) but not white (264 of 826 [32.0%]; AOR, 1.16; 95% CI, 0.93-1.44; *P* = .20) standardized packaging compared with the fully branded packaging (238 of 828 [28.7%]). Adults had lower odds of reporting no interest in trying e-cigarettes in green standardized packaging (3505 of 4040 [86.8%]; AOR, 0.85; 95% CI, 0.73-0.99; *P* = .046) but not white packaging (3532 of 4006 [88.2%]; AOR, 1.05; 95% CI, 0.89-1.23; *P* = .59) compared with branded packaging (3526 of 4000 [88.1%]). Youths who had never vaped (275 of 699 [39.3%]; AOR, 1.34; 95% CI, 1.07-1.69; *P* = .01) and youths who had never smoked (271 of 676 [40.1%]; AOR, 1.38; 95% CI, 1.10-1.75; *P* = .006) were more likely to report no interest in trying e-cigarettes in green packaging compared with branded packaging (224 of 688 [32.6%] never vaping; 216 of 662 [32.6%] never smoking). There were no significant differences by vaping or smoking status among adults.

**Conclusions and Relevance:**

The findings of this survey study suggest that standardized packaging measures may reduce the appeal of e-cigarettes among youths without reducing their appeal among adults.

## Key Points

**Question:**

Is there an association between standardized e-cigarette packaging and interest in trying e-cigarette products among samples of youths and adults in Great Britain?

**Findings:**

In this study comprising 2 surveys conducted among 2469 youths (aged 11-18 years) and 12 046 adults (aged ≥18 years) from Great Britain, youths had higher odds of reporting no interest in trying e-cigarettes in standardized green packaging than e-cigarettes in branded packaging, but adults had lower odds of reporting no interest in trying e-cigarettes in standardized green packaging than e-cigarettes in branded packaging.

**Meaning:**

This study suggests that standardized packaging measures may reduce the appeal of e-cigarettes among youths without reducing their appeal among adults.

## Teaser

This survey study examines the association of fully branded vs standardized e-cigarette packaging with interest in trying products among youths and adults in Great Britain.

## Introduction

In Great Britain in 2022, 8.3% of adults and 7.0% of youths aged 11 to 17 years reported current vaping.^1,2^ Vaping products in Great Britain can legally be sold only to those aged 18 years or older.^3^ Vaping can help some smokers to quit smoking.^4,5^ However, there is concern about youths’ use of vaping products, particularly with the recent increase in the use of disposable vaping products among youths younger than 18 years in Great Britain.^6^

e-Cigarette marketing, including packaging, has been found to influence the appeal of vaping products to youth and adult smokers.^7,8^ e-Cigarette packaging varies substantially, with several brands featuring vibrant colors to promote products.^9,10^ Such marketing techniques are the same as those previously used by tobacco companies; marketing via packaging is particularly prominent in jurisdictions where marketing via other forms, such as mass media, has been restricted.^11,12^

Standardized (or “plain”) packaging for combustible tobacco cigarettes was introduced in Great Britain in May 2016, requiring cigarettes and rolling tobacco to be manufactured and sold in standardized Pantone 448 C olive-green packs with a matte finish and the brand name in standard font and no brand imagery or logos.^3^ In Great Britain, e-cigarette regulations are less restrictive than tobacco restrictions and do not mandate standardized packaging. However, e-cigarette regulations include restrictions on promotion^13^ and require packaging to include nicotine content, hazard symbols, and a health warning about nicotine.^3^

Standardized tobacco packaging is effective for reducing the appeal of tobacco cigarettes, particularly among young people.^14,15^ However, there is a lack of evidence on whether standardized e-cigarette packaging would influence the appeal of e-cigarettes and whether this influence would vary between youth and adult populations. This is an important issue in Great Britain, where the public policy position is that adult smokers’ use of e-cigarettes as a smoking cessation aid should be encouraged but use among youths should be discouraged.^16^ Research is therefore needed to evaluate the potential association of standardized packaging of e-cigarettes with interest in trying the product among both youths and adults who do or do not smoke. Using an experimental design, this survey study aimed to examine associations between standardized e-cigarette packaging and e-cigarette product appeal among samples of youths and adults in Great Britain.

## Methods

### Data Sources

The data provided in this survey study were obtained from 2 online surveys: the Action on Smoking and Health (ASH) 2021 Smokefree Great Britain Youth Survey and the ASH 2021 Smokefree Great Britain Adult Survey. For the youth survey, informed consent was provided either by the parents of those aged 11 to 15 years or by individuals aged 16 to 18 years. For the adult survey, informed consent was provided by participants. Ethical approval for the analyses in this study was not required because this study involved secondary analysis of preexisting data, in line with King’s College London policy. This study followed the American Association for Public Opinion Research (AAPOR) reporting guideline.

#### ASH Youth Survey

The online 2021 ASH Smokefree Great Britain Youth Survey collected data from young people aged 11 to 18 years between March 25 and April 16, 2021. Participants were drawn from an existing online panel maintained by YouGov. Active sampling was used, which dynamically evaluates what surveys are available for a particular panel member based on their personal characteristics, and restrictions are put in place to ensure that only those who are selected from their panel of registered users are allowed to take part in the survey.^17^

The experiment was conducted as part of the online ASH Smokefree Great Britain Youth Survey. A between-individuals experimental design was used to examine perceptions of e-cigarette packs that were digitally altered to remove brand imagery and color (Figure). Participants were assigned to 1 of 3 experimental conditions using simple randomization at a ratio of 1:1:1. Within each condition, participants viewed a set of the same 3 brands of e-cigarette starter kits (device and cartridges), 2 of which were pod devices and the third a tank device. The 3 conditions included images of fully branded packaging (control), images of white standardized e-cigarette packaging, and images of green standardized e-cigarette packaging (Figure).

**Figure.**
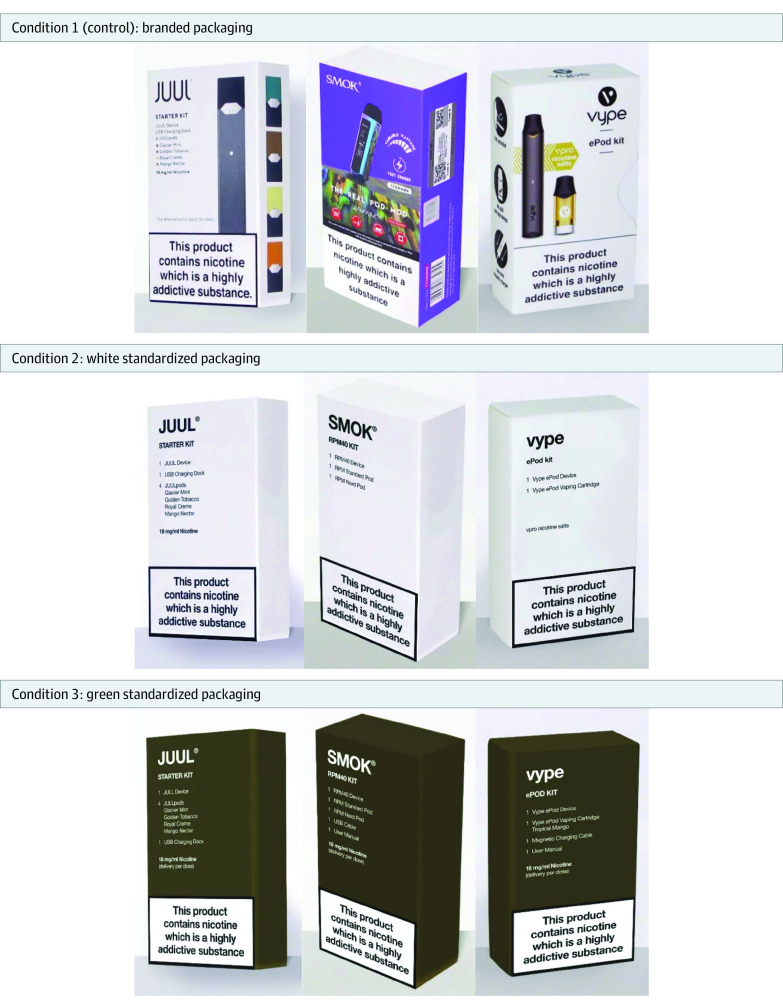
e-Cigarette Packs by Experimental Condition

The green standardized pack color, using the same olive-green Pantone 448 C shade in a matte finish, was chosen to reflect the current standardized packaging requirements for tobacco cigarette packs in Great Britain.^3^ The white standardized pack color was chosen by considering evidence showing that tobacco cigarettes displayed in lighter colored packs were perceived to be less harmful.^18^ The brands presented to participants were the most popular brands in Great Britain at the time of the study.^19^

The survey was completed by 2513 youths. Respondents who reported “Prefer not to say” for the outcome (n = 13) or any covariates (n = 31) were excluded from the sample, resulting in a final analytic sample of 2469 respondents. eTable 1 in Supplement 1 lists the measures used and their coding. The sociodemographic covariates were sex (male or female), age group (11-15 years and 16-18 years), and socioeconomic status (ABC1 [high socioeconomic status; intermediate and higher managerial, administrative, supervisory, clerical, and junior managerial professional occupations] and C2DE [low socioeconomic status; skilled, semi-skilled, and unskilled manual occupations; unemployed; and lowest-grade occupations]; eTable 2 in Supplement 1).

##### No Interest in Trying e-Cigarette Products Among People Your Age (Outcome)

Respondents were shown a set of 3 e-cigarette packs based on experimental condition (Figure) and asked, “Which of the following products would people your age be most interested in trying?” Response options included “None of these products,” “I don’t know,” and “Prefer not to say.” Responses were dichotomized as “None of these products” vs any other response. Respondents who selected “Prefer not to say” were excluded.

##### Vaping Status

Respondents were asked, “Have you ever heard of e-cigarettes? They are also sometimes called vapes, shisha pens or electronic cigarettes.” Those who responded “Yes” were asked “Which ONE of the following is closest to describing your experience of e-cigarettes?” with available responses ranging from “I have never used an e-cigarette” to “I use e-cigarettes every day.” Response options were classified into 3 categories: “never user,” “ever user,” and “current user” (including occasional and regular vaping) (eTable 1 in Supplement 1).

##### Smoking Status

Respondents were asked which statement best applied to their experience with cigarettes. Response options were classified into 3 categories: “never smoker,” “ever smoker,” and “current smoker” (including occasional and regular smoking) (eTable 1 in Supplement 1).

#### ASH Adult Survey

The online 2021 ASH Smokefree Great Britain Adult Survey collected data on tobacco and vaping product use among adults aged 18 years or older between February 18 and March 18, 2021. Like the ASH Youth Survey, participants were drawn from an existing online panel maintained by YouGov using active sampling. The experimental design and 3 conditions used in the ASH Adult Survey were identical to those used in the ASH Youth Survey (Figure).

The survey was completed by 12 248 adults. Respondents who reported “Prefer not to say” (n = 47) for the outcome measure and/or “Don’t know” for covariates (n = 155) were excluded, resulting in a final analytic sample of 12 046 respondents. eTable 1 in Supplement 1 lists the measures used and their coding. The sociodemographic covariates were sex (male or female), age group (18-29, 30-39, 40-49, 50-59, and ≥60 years), and socioeconomic status (ABC1, C2DE; see eTable 2 in Supplement 1).

##### No Interest in Trying e-Cigarette Products (Outcome)

Respondents were shown a set of 3 e-cigarette packs based on experimental condition (Figure) and asked, “Which of the following products would you be most interested in trying?” Like the ASH Youth survey, response options were dichotomized as “None of these products” vs any other response. The wording of the outcome measure differed between youths and adults, such that the youth measure asked about interest in trying e-cigarette products among “people your age,” whereas the adult measure asked about respondents’ own interest in trying the product. Questions were worded differently for youths and adults due to concerns about asking youths—particularly those who had never vaped—whether they would be interested in trying a product that they may not have been exposed to prior to this study.

##### Vaping Status

Respondents were asked which statement best described their experience with e-cigarettes: “I have never heard of e-cigarettes and never tried them,” “I have heard of e-cigarettes but have never tried them,” “I have tried e-cigarettes but do not use them (anymore),” “I have tried e-cigarettes but still use them,” and “Don’t know.” Ever vapers were subsequently asked how often they had used or currently used e-cigarettes, with response options ranging from “Less than once a month” to “Every day.” Responses were classified into 3 categories: “never vapers,” “ever vapers,” and “current vapers” (including occasional and regular vaping) (eTable 1 in Supplement 1).

##### Smoking Status

Respondents were asked which statement best applied to their experience with cigarettes. Response options were classified into 3 categories: “never smokers,” “former smokers,” and “current smokers” (including occasional and regular smoking) (eTable 1 in Supplement 1).

### Statistical Analysis

The χ^2^ test was used to test for successful randomization to conditions (eTables 2 and 3 in Supplement 1). First, a logistic regression model was fit to examine whether no interest in trying any e-cigarette products differed between the branded pack condition (reference group) and the green or white standardized pack conditions, adjusting for sex, age group, social grade, vaping status, and smoking status. Second, 2-way interactions between (1) pack condition and vaping status and (2) pack condition and smoking status were tested to examine potential subgroup differences. Third, sensitivity analyses used multinomial regression to examine whether differences were observed in model results when including the “I don’t know” response option as a separate category. Separate models were estimated for the youth and adult samples.

All analyses were conducted in SPSS, version 28 (IBM Corp). All statistical tests were 2-sided, and *P* < .05 was considered statistically significant.

## Results

The ASH Youth Survey was completed by 2513 youths, of whom 2469 (1286 female youths [52.1%]; mean [SD] age, 15.0 [2.3] years) were included in the analytical sample; the ASH Adult Survey was completed by 12 248 adults, of whom 12 046 (6412 women [53.2%]; mean [SD] age, 49.9 [17.4] years) were included in the analytical sample. Participant demographic characteristics are presented in eTables 3 and 4 in Supplement 1. For youths, there were similar proportions of female (1286 [52.1%) and male (1183 [47.9%]) participants, a higher proportion of respondents from a high socioeconomic background (ABC1; 1748 [70.8%]) than low socioeconomic background (C2DE; 721 [29.2%]), and most participants had never smoked (2024 [82.0%]) or vaped (2085 [84.4%]). Similar characteristics were observed among adult participants.

When testing for successful randomization, except for social grade among the adult sample (χ^2^ = 8.44; *P* = .01), no significant differences in sociodemographic characteristics or vaping or smoking status were observed across youth or adult experimental conditions.

### Packaging Experiment: Youth Survey

Overall (across all conditions), 943 youths (38.2%) reported that people their age would be interested in trying any of the vaping products, 794 (32.2%) reported that people their age would have no interest in trying any of the products, and 732 (29.6%) reported that they did not know which of the displayed vaping products people their age would be most interested in trying (eTable 5 in Supplement 1). Table 1 shows the associations between reporting no interest among people their age in trying any of the products shown and packaging condition among youths. Youths who were shown green standardized packaging (292 of 815 [35.8%]; adjusted odds ratio [AOR], 1.37; 95% CI, 1.10-1.71; *P* = .005), but not white standardized packaging (264 of 826 [32.0%]; AOR, 1.16; 95% CI, 0.93-1.44; *P* = .20), were statistically significantly more likely to report no interest compared with those shown the branded packaging (238 of 828 [28.7%]). There was no significant difference in reporting no interest between youths who were shown green standardized packaging (292 of 815 [35.8%]) and youths who were shown white standardized packaging (264 of 826 [32.0%]; AOR, 1.17; 95% CI, 0.94-1.48; *P* = .16).

**Table 1.** Adjusted Associations Between Reporting No Interest Among People Their Age in Trying Any of the Products Shown and Packaging Condition, ASH Youth Survey 2021 (N = 2469)^a^

Characteristic	No interest in trying any products among people their age		*P* value
	No. (%)	AOR (95% CI)	
Total	794 (32.2)	NA	NA
Packaging condition			
Branded	238/828 (28.7)	1 [Reference]^b^	NA
Green standardized	292/815 (35.8)	1.37 (1.10-1.71)	.005
White standardized	264/826 (32.0)	1.16 (0.93-1.44)	.20
Sex			
Male	408/1183 (34.5)	1 [Reference]	NA
Female	386/1286 (30.0)	0.85 (0.71-1.01)	.07
Socioeconomic status^c^			
C2DE	241/721 (33.4)	1 [Reference]	NA
ABC1	553/1748 (31.6)	1.08 (0.88-1.33)	.48
Age group, y			
11-15	588/1330 (44.2)	1 [Reference]	NA
16-18	206/1139 (18.1)	0.33 (0.27-0.40)	<.001
Vaping status			
Never^d^	750/2085 (36.0)	1 [Reference]	NA
Ever	37/271 (13.7)	0.52 (0.34-0.78)	.001
Current	7/113 (6.2)	0.24 (0.10-0.55)^e^	.001
Smoking status			
Never	728/2024 (36.0)	1 [Reference]	NA
Former	55/321 (17.1)	0.71 (0.50-1.01)	.06
Current	11/124 (8.9)	0.51 (0.25-1.03)^e^	.06

Abbreviations: AOR, adjusted odds ratio; ASH, Action on Smoking and Health; NA, not applicable.

^a^ Analyses were adjusted for sex, age group, socioeconomic status, vaping status, and smoking status. All data are unweighted.

^b^ Reference category is selecting any of the products or “Don’t know.”

^c^ ABC1 indicates high socioeconomic status: intermediate and higher managerial, administrative, supervisory, clerical, and junior managerial professional occupations. C2DE indicates low socioeconomic status: skilled, semi-skilled, and unskilled manual occupations; unemployed; and lowest-grade occupations.

^d^ Includes respondents who had never heard of e-cigarettes.

^e^ Estimates likely unreliable due to low sample sizes.

A significantly smaller percentage of youths aged 16 to 18 years reported no interest in trying any of the products compared with youths aged 11 to 15 years. A significantly smaller percentage of youths who currently vaped or had ever vaped reported no interest in trying any of the products compared with youths who had never vaped. The percentage reporting no interest in trying any of the products was higher among youths who had never smoked than those who had ever or were currently smoking; however, these comparisons were not significant (Table 1).

Because a substantial proportion of youths reported that they did not know which of the displayed vaping products people their age would be interested in trying, a sensitivity analysis was conducted. A multinomial regression model was run in which “Don’t know” was treated as a separate category in the outcome variable. When “Don’t know” was a separate category, youths were more likely to report no interest among people their age in trying products in either green (292 of 815 [35.8%]) or white (264 of 826 [32.0%]) standardized packs compared with branded packs (238 of 828 [28.7%]) (eTable 5 in Supplement 1). Youths were also more likely to report that they did not know which product people their age would be most interested in when products were shown in either green (251 of 815 [30.8%]) or white (250 of 826 [30.3%]) standardized packs compared with branded packs (231 of 828 [27.9%]).

Interactions were explored between vaping status and packaging condition (eFigure 1 and eTable 6 in Supplement 1). Contrasts of packaging conditions by vaping status indicated that youths who had never vaped were more likely to report no interest in trying e-cigarette products in green packaging (275 of 699 [39.3%]; AOR, 1.34; 95% CI, 1.07-1.69; *P* = .01) compared with branded packaging (224 of 688 [32.6%]). There were no significant differences when contrasting packaging conditions within ever vapers and current vapers (eTable 6 in Supplement 1).

Interactions were also explored between smoking status and packaging condition (eFigure 2 and eTable 6 in Supplement 1). Contrasts of packaging conditions by smoking status indicated that youths who had never smoked were more likely to report no interest in trying e-cigarette products in green packaging (271 of 676 [40.1%]; AOR, 1.38; 95% CI, 1.10-1.75; *P* = .006) compared with branded packaging (216 of 662 [32.6%]). There were no significant differences when contrasting packaging conditions within ever smokers and current smokers (eTable 6 in Supplement 1).

### Packaging Experiment: Adult Survey

Overall, 947 adults (7.9%) reported that they were interested in trying any of the vaping products, 10 563 (87.7%) reported no interest in trying any of the products, and 536 (4.4%) reported that they did not know which of the displayed vaping products they would be most interested in trying (eTable 7 in Supplement 1).

Table 2 shows the associations between reporting no interest in trying any of the products shown and packaging conditions among adults. There were no significant differences in reporting no interest among adults who were shown products in standardized white packaging (3532 of 4006 [88.2%]; AOR, 1.05; 95% CI, 0.89-1.23; *P* = .59) compared with branded packaging (3526 of 4000 [88.1%]). However, adults were significantly less likely to report no interest in trying products shown in green packaging (3505 of 4040 [86.8%]; AOR, 0.85; 95% CI, 0.73-0.99; *P* = .046) compared with branded packaging (3626 of 4006 [88.1%]). Adults were also significantly less likely to report no interest in trying products in green packaging (3505 of 4040 [86.8%]) compared with products in white packaging (3532 of 4006 [88.2%]; AOR, 0.81; 95% CI, 0.70-0.95; *P* = .01).

**Table 2.** Associations Between Reporting No Interest in Trying Any of the Products Shown and Packaging Condition, ASH Adult Survey 2021 (N = 12 046)^a^

Characteristic	No interest in trying any products		*P* value
	No. (%)	AOR (95% CI)	
Total	10 563 (87.7)	NA	NA
Packaging condition			
Branded	3526/4000 (88.1)	1 [Reference]^b^	NA
Green standardized	3505/4040 (86.8)	0.85 (0.73-0.99)	.046
White standardized	3532/4006 (88.2)	1.05 (0.89-1.23)	.59
Sex			
Male	4882/5634 (86.7)	1 [Reference]	NA
Female	5681/6412 (88.6)	1.07 (0.94-1.21)	.34
Age group, y			
18-29	1598/1968 (81.2)	1 [Reference]	NA
30-39	1577/1910 (82.6)	1.64 (1.34-2.01)	<.001
40-49	1729/2040 (84.8)	1.85 (1.50-2.27)	<.001
50-59	1633/1818 (89.8)	2.84 (2.25-3.59)	<.001
≥60	4026/4310 (93.4)	4.07 (3.31-5.01)	<.001
Socioeconomic status^c^			
C2DE	4320/5002 (86.4)	1 [Reference]	NA
ABC1	6243/7044 (88.6)	1.11 (0.97-1.27)	.13
Vaping status			
Never^d^	9156/9587 (95.5)	1 [Reference]	NA
Ever	740/1082 (68.4)	0.29 (0.24-0.35)	<.001
Current	667/1377 (48.4)	0.09 (0.08-0.11)	<.001
Smoking status			
Never	6139/6395 (96.0)	1 [Reference]	NA
Former	3616/4191 (86.3)	0.48 (0.39-0.58)	<.001
Current	808/1460 (55.3)	0.15 (0.12-0.18)	<.001

Abbreviations: AOR, adjusted odds ratio; ASH, Action on Smoking and Health; NA, not applicable.

^a^ Analyses were adjusted for sex, age group, socioeconomic status, vaping status, and smoking status. All data are unweighted.

^b^ Reference category is “Other,” including selecting any of the products and “Don’t know.”

^c^ ABC1 indicates high socioeconomic status: intermediate and higher managerial, administrative, supervisory, clerical, and junior managerial professional occupations. C2DE indicates low socioeconomic status: skilled, semi-skilled, and unskilled manual occupations; unemployed; and lowest-grade occupations.

^d^ Includes respondents who had never heard of e-cigarettes.

The percentage of adults reporting no interest was higher among all older age groups compared with respondents aged 18 to 29 years and was lower among current vapers than never vapers and ever vapers, and among current smokers and former smokers than never smokers (Table 2).

With respect to the sensitivity analyses, when “Don’t know” was included as its own category in multinomial regression, adults were more likely to report no interest in trying products in white packaging compared with branded packaging. Adults were also more likely to report that they did not know which product they were most interested in trying when products were shown in green or white packaging compared with branded packaging (eTable 8 in Supplement 1).

Interactions were explored within vaping status and packaging condition (eFigure 3 in Supplement 1) and within smoking status and packaging condition (eFigure 4 and eTable 8 in Supplement 1). There were no significant differences in interest in trying any of the products when contrasting packaging conditions by vaping status or by smoking status (eTable 8 in Supplement 1).

## Discussion

Findings differed between youths and adults in the associations between e-cigarette packaging and interest in trying e-cigarette products. Compared with fully branded packaging, green standardized e-cigarette packaging with no brand imagery was associated with decreased interest in trying the vaping products shown among youths but not adults. However, there was no significant difference in interest in trying e-cigarettes in white standardized packaging with no brand imagery compared with branded packaging among either youths or adults.

For youths, our overall findings are consistent with previous studies that found that green standardized packaging of tobacco cigarettes reduces their appeal.^14,15^ Compared with branded packaging, white standardized packaging was also associated with lower appeal among youths when sensitivity analyses accounted for “Don’t know” responses. Furthermore, a greater proportion of youths who had never smoked or never vaped perceived no interest among people their age in trying any of the e-cigarette products shown in green standardized packaging compared with the branded packaging condition. Therefore, green standardized packaging may deter the use of e-cigarettes among youths who do not already vape or smoke. However, there were too few ever and current vaping youths and ever and current smoking youths to reliably examine the associations of standardized packaging among these groups.

Among adults, the level of no interest in trying any of the e-cigarette products shown was similar across branded, green standardized, and white standardized packaging; however, no interest in trying the products was significantly lower among those exposed to green standardized packaging than branded or standardized white packaging, and sensitivity analyses suggested this was due to an increase in “Don’t know” responses for green standardized packaging. Interest by product packaging also did not differ by vaping and smoking status among adults; thus, our research suggests that removing branded elements from packaging would most likely not impact adult smokers’ interest in trying vaping products. Future research should examine the association of standardized packaging with perceptions of vaping, specifically perceptions of vaping as effective smoking cessation.

### Limitations and Strengths

This study has some limitations. First, there were differences between the youth and adult surveys in the wording of the items assessing outcomes, as well as vaping and smoking status. The ASH Youth Survey measure asked respondents about interest among people their age; therefore, responses did not represent participants’ own interest in trying the products shown, but rather their perception of peer interest. However, the ASH Adult Survey measure asked about respondents’ own interest in trying the products shown, meaning that questions for youths and adults were measuring slightly different concepts. Hence, it was not appropriate to directly test for differences between the youth and adult responses. Second, our samples were limited to Great Britain, so findings cannot be generalized to other countries, particularly those with different regulations on e-cigarette and cigarette packaging.

This study also has some strengths, including analysis of the associations between standardized e-cigarette packaging and interest. Findings therefore offer valuable and timely evidence that can be used to inform the development of e-cigarette packaging regulations.

## Conclusions

The findings of this survey study suggest that reducing brand imagery through standardized e-cigarette packaging is associated with decreased appeal of e-cigarette products among youths, specifically never smokers and never vapers, without reducing its appeal among adult smokers. Overall, our findings lend support for reducing brand imagery on e-cigarette products in Great Britain.

## Notes

Supplement 1. **eTable 1**. Measure Wording for Outcome, Vaping Status, and Smoking Status in the ASH Youth and Adult Surveys **eTable 2.** Social Grade Classification System, Based on Occupation of the Chief Income Earner in the Household **eTable 3.** Sample Characteristics by Experimental Packaging Condition, 2021 ASH Smokefree GB Youth Survey (N=2469) **eTable 4.** Sample Characteristics by Experimental Packaging Condition, 2021 ASH Smokefree GB Adult Survey (n=12046) **eTable 5.** Multinomial Associations Between Reporting No Interest in Trying or “Don’t Know" and Packaging Condition, 2021 ASH Smokefree GB Youth Survey (n=2469) **eTable 6.** Interactions Between Packaging Condition and Vaping and Smoking Status, 2021 ASH Smokefree GB Youth Survey (n=2469) **eFigure 1.** No Interest in Trying Vaping Product by Vaping Status, ASH Youth (n=2469) **eFigure 2.** No Interest in Trying Vaping Product by Smoking Status, ASH Youth (n=2469) **eTable 7.** Multinomial Associations Between Reporting No Interest or Don’t Know and Packaging Condition, 2021 ASH Smokefree GB Adult Survey (n=12046) **eTable 8.** Interactions Between Packaging Condition and Vaping and Smoking Status, 2021 ASH Smokefree GB Adult Survey (n=12046) **eFigure 3.** No Interest in Trying Vaping Product by Vaping Status, ASH Adult (n=12046) **eFigure 4.** No Interest in Trying Vaping Product by Smoking Status, ASH Adult (n=12046)

Supplement 2.
Data Sharing Statement
