# Supplementary material for: Association of Fully Branded and Standardized e-Cigarette Packaging With Interest in Trying Products Among Youths and Adults in Great Britain
Source: JAMA Netw Open. 2023 Mar 14;6(3):e231799. doi: 10.1001/jamanetworkopen.2023.1799 (PMC10015302; doi:10.1001/jamanetworkopen.2023.1799)
Supplement: Supplement 1. — eTable 1. Measure Wording for Outcome, Vaping Status, and Smoking Status in the ASH Youth and Adult Surveys eTable 2. Social Grade Classification System, Based on Occupation of the Chief Income Earner in the Household eTable 3. Sample Characteristics by Experimental Packaging Condition, 2021 ASH Smokefree GB Youth Survey (N=2469) eTable 4. Sample Characteristics by Experimental Packaging Condition, 2021 ASH Smokefree GB Adult Survey (n=12046) eTable 5. Multinomial Associations Between Reporting No Interest in Trying or “Don’t Know" and Packaging Condition, 2021 ASH Smokefree GB Youth Survey (n=2469) eTable 6. Interactions Between Packaging Condition and Vaping and Smoking Status, 2021 ASH Smokefree GB Youth Survey (n=2469) eFigure 1. No Interest in Trying Vaping Product by Vaping Status, ASH Youth (n=2469) eFigure 2. No Interest in Trying Vaping Product by Smoking Status, ASH Youth (n=2469) eTable 7. Multinomial Associations Between Reporting No Interest or Don’t Know and Packaging Condition, 2021 ASH Smokefree GB Adult Survey (n=12046) eTable 8. Interactions Between Packaging Condition and Vaping and Smoking Status, 2021 ASH Smokefree GB Adult Survey (n=12046) eFigure 3. No Interest in Trying Vaping Product by Vaping Status, ASH Adult (n=12046) eFigure 4. No Interest in Trying Vaping Product by Smoking Status, ASH Adult (n=12046) [file jamanetwopen-e231799-s001.pdf]

Supplementary Online Content

Taylor E, Arnott D, Cheeseman H, et al. Association of fully branded and standardized e-cigarette packaging with interest in trying products among youths and adults in Great Britain. *JAMA Netw Open.* 2023;6(3):e231799. doi:10.1001/jamanetworkopen.2023.1799

eTable 1. Measure Wording for Outcome, Vaping Status, and Smoking Status in the ASH Youth and Adult Surveys
eTable 2. Social Grade Classification System, Based on Occupation of the Chief Income Earner in the Household
eTable 3. Sample Characteristics by Experimental Packaging Condition, 2021 ASH Smokefree GB Youth Survey (N=2469)
eTable 4. Sample Characteristics by Experimental Packaging Condition, 2021 ASH Smokefree GB Adult Survey (n=12046)
eTable 5. Multinomial Associations Between Reporting No Interest in Trying or "Don't Know" and Packaging Condition, 2021 ASH Smokefree GB Youth Survey (n=2469)
eTable 6. Interactions Between Packaging Condition and Vaping and Smoking Status, 2021 ASH Smokefree GB Youth Survey (n=2469)
eFigure 1. No Interest in Trying Vaping Product by Vaping Status, ASH Youth (n=2469)
eFigure 2. No Interest in Trying Vaping Product by Smoking Status, ASH Youth (n=2469)
eTable 7. Multinomial Associations Between Reporting No Interest or Don't Know and Packaging Condition, 2021 ASH Smokefree GB Adult Survey (n=12046)
eTable 8. Interactions Between Packaging Condition and Vaping and Smoking Status, 2021 ASH Smokefree GB Adult Survey (n=12046)
eFigure 3. No Interest in Trying Vaping Product by Vaping Status, ASH Adult (n=12046)
eFigure 4. No Interest in Trying Vaping Product by Smoking Status, ASH Adult (n=12046)

This supplementary material has been provided by the authors to give readers additional information about their work.

© 2023 Taylor E et al. *JAMA Network Open.*

**eTable 1**. Measure Wording for Outcome, Vaping Status, and Smoking Status in the ASH Youth and Adult Surveys	
ASH Youth	ASH Adult
NO INTEREST IN TRYING PRODUCTS SHOWN (OUTCOME)	
‘Which of the following products would *people your age* be most interested in trying?’<br>a‘JUUL’<br>b‘Smok’<br>c‘Vype’<br>‘Prefer not to say’ (excluded)<br>e‘None of these products’<br>f‘Don’t know’<br><br>Coding<br>No interest in trying any product (e) vs. Other (a-c, f)	‘Which of the following products would you be most interested in trying?’<br>a‘JUUL’<br>b‘Smok’<br>c‘Vype’<br>‘Prefer not to say’ (excluded)<br>e‘I have no interest in trying any of these products’<br>f‘Don’t know’<br><br>Coding<br>No interest in trying any product (e) vs. Other (a-c, f)
VAPING STATUS	
1. ‘Have you ever heard of e-cigarettes? They are also sometimes called vapes, shisha pens or electronic cigarettes.’<br><br>a‘Yes’<br>b‘No’<br>c‘Don’t know’<br><br>2: *Among those who had ever heard of e-cigarettes:* ‘Which ONE of the following is closest to describing your experience of e-cigarettes?’<br><br>a‘I have never used an e-cigarette’<br>b‘I have only tried an e-cigarette once or twice’<br>c‘I use e-cigarettes sometimes, but no more than once a month’<br>d‘I use e-cigarettes more than once a month, but less than once a week’<br>e‘I use e-cigarettes more than once a week but not every day’<br>f‘I use e-cigarettes every day’<br>g‘I used e-cigarettes in the past but no longer do’<br>‘Don’t want to say’(excluded)<br><br>Coding<br>Never (1b, 1c, 2a), Ever (2b, 2g), Current (2c-2f)	‘E-cigarettes are also sometimes called vapes or vaping devices. Which of the following statements BEST applies to you?’<br><br>a‘I have never heard of e-cigarettes and have never tried them’<br>b‘I have heard of e-cigarettes but have never tried them’.<br>c’I have tried e-cigarettes but do not use them (anymore)’<br>d’I have tried e-cigarettes and still use them<br>’Don’t know (excluded)<br><br>2. *Among ever users* (1c, 1d): ‘You told us that you either used to use or still use e-cigarettes…How OFTEN did you use/ do you currently use e-cigarettes?’<br><br>a‘Everyday’<br>b‘A few times a week’<br>c‘Once a week’<br>d‘Once or twice a month’<br>e‘Less than once a month’<br>f‘Don’t know/ can’t remember’<br>g‘Not applicable – I have only tried e-cigarettes once or twice’<br><br>Coding<br>Never (1a,1b), Ever (2e, 2f, 2g), Current (2a-2d)
SMOKING STATUS	
’Which ONE of the following BEST applies to you?’<br>a‘I have never smoked cigarettes, not even a puff or two’<br>b‘I have only ever tried smoking cigarettes once’<br>c‘I used to smoke sometimes but I never smoke cigarettes now’<br>d‘I sometimes smoke cigarettes now but less than once a week’<br>e‘I usually smoke between once and six cigarettes a week’<br>f‘I usually smoke more than six cigarettes a week’<br>‘Don’t want to say’(excluded)<br><br>Coding: Never (a), Ever (b, c), Current (d-f)	‘Smoking in this survey refers to all burnt tobacco products. It does NOT include e-cigarettes. Which of the following statements BEST applies to you?’<br><br>a‘I have never smoked’<br>b‘I used to smoke but I have given up now’<br>c‘I smoke but don’t smoke every day’<br>d‘I smoke every day’<br><br>Coding<br>Never (a), Former (b), Current (c-d)

© 2023 Taylor E et al. *JAMA Network Open.*

eTable 2. Social Grade Classification System, Based on Occupation of the Chief Income Earner in the Household

A	Higher managerial, administrative and professional
B	Intermediate managerial, administrative and professional
C1	Supervisory, clerical and junior managerial, administrative and professional
C2	Skilled manual workers
D	Semi-skilled and unskilled manual workers
E	State pensioners, casual and lowest grade workers, unemployed with state benefits only

For ASH-Youth: social grade was based on the occupation of the chief income earner in the household, and was asked of the parents of those participants age 11–15, and directly of those participants age 16–18.

For ASH-Adults: social grade was based on the occupation of the chief income earner in the household, and was directly asked to participants.

© 2023 Taylor E et al. *JAMA Network Open.*

eTable 3. Sample Characteristics by Experimental Packaging Condition, 2021 ASH Smokefree GB Youth Survey (N=2469)

		Experimental Packaging Condition			
		Branded (n=828)	Green Standardised (n=815)	White Standardised (n=826)	χ^2 [c]
		%(N)	%(N)	%(N)	χ^2(p)
Sex	Female	53.1(440)	50.6(412)	52.5(434)	1.21(.55)
	Male	46.9(388)	49.4(403)	47.5(392)	
Age group	11-15 years	53.7(445)	54.2(442)	53.6(443)	0.67(.97)
	16-18 years	46.3(383)	45.8(373)	46.4(383)	
Social Grade [a]	ABC1	71.0(588)	68.8(561)	72.5(599)	2.17(.26)
	C2DE	29.0(240)	31.2(254)	27.5(227)	
Vaping status	Never [b]	83.1(688)	85.8(699)	84.5(698)	5.70(.22)
	Ever	11.8(98)	10.9(89)	10.2(84)	
	Current	5.1(42)	3.3(27)	5.3(44)	
Smoking status	Never	80.0(662)	82.9(676)	83.1(686)	5.47(.24)
	Ever	14.6(121)	12.9(105)	11.5(95)	
	Current	5.4(45)	4.2(34)	5.4(45)	

[a] ABC1: higher and intermediate managerial, administrative, supervisory, clerical and junior managerial, professional occupations; C2DE: skilled, semi-skilled and unskilled manual occupations, unemployed and lowest grade occupations.

[b] Includes respondents who had never heard of e-cigarettes.

[c] A Chi-Square Test was performed to determine whether the randomisation to experimental conditions was equal across demographic groups.

Note: Estimates shown above are based on the unweighted sample.

© 2023 Taylor E et al. *JAMA Network Open.*

eTable 4. Sample Characteristics by Experimental Packaging Condition, 2021 ASH Smokefree GB Adult Survey (n=12046)

		Experimental Packaging Condition			
		Branded Packs (n=4000)	Green Standardised Packs (n=4040)	White Standardised Packs (n=4006)	χ^2 [c]
		%(N)	%(N)	%(N)	χ^2(p)
Sex	Male	47.2(1886)	46.2(1865)	47.0(1883)	0.92(.63)
	Female	52.9(2114)	53.8(2175)	53.0(2123)	
Age group	18-29	15.7(629)	16.6(670)	16.7(669)	11.69(.20)
	30-39	15.3(612)	16.0(646)	16.3(652)	
	40-49	18.1(722)	16.2(654)	16.6(664)	
	50-59	14.5(581)	15.0(607)	15.7(630)	
	≥60	36.4(1456)	36.2(1463)	34.7(1391)	
Social Grade [a]	C2DE	43.0(1721)	41.7(1685)	39.8(1596)	**8.44(.02)**
	ABC1	57.0(2279)	58.3(2355)	60.2(2410)	
Vaping status	Never [b]	79.9(3197)	79.7(3220)	79.1(3170)	1.81(.77)
	Ever	8.6(342)	9.0(365)	9.4(375)	
	Current	11.5(461)	11.3(455)	11.5(461)	
Smoking status	Never	53.2(2127)	52.8(2135)	53.2(2133)	0.26(.99)
	Former	34.7(1388)	34.9(1409)	34.8(1394)	
	Current	12.1(485)	12.3(496)	12.0(479)	

[a] ABC1: higher and intermediate managerial, administrative, supervisory, clerical and junior managerial, professional occupations; C2DE: skilled, semi-skilled and unskilled manual occupations, unemployed and lowest grade occupations.
[b] Includes respondents who had never heard of e-cigarettes.
[C] A Chi-Square Test was performed to determine whether the randomisation to experimental conditions was equal across demographic groups.
Note: estimates shown above are based on the unweighted sample.
Bold indicate significance to p<.05

© 2023 Taylor E et al. *JAMA Network Open.*

**eTable 5.** Multinomial Associations Between Reporting No Interest in Trying or "Don't Know" and Packaging Condition, 2021 ASH Smokefree GB Youth Survey (n=2469)							
	Selected a product (Ref)	No interest in trying any product shown			Don't know		
	%(n)	%(n)	AOR(95%CI)	p	%(n)	AOR(95%CI)	p
**Total**	38.2(943)	32.2(794)			29.6(732)		
**Packaging condition**							
Branded 'Control'	43.4(359)	28.7(238)	Ref		27.9(231)	Ref	
Green Standardised	33.4(272)	35.8(292)	1.68(1.30-2.17)	<.001	30.8(251)	1.47(1.14-1.89)	.003
White Standardised	37.8(312)	32.0(264)	1.32(1.02-1.70)	.03	30.3(250)	1.29(1.01-1.65)	<.05
**Sex**							
Male	35.0(414)	34.5(408)	Ref		30.5(361)	Ref	
Female	41.1(529)	30.0(386)	0.76(0.62-0.94)	.01	28.8(371)	0.81(0.66-0.99)	.<.05
**Socioeconomic status**							
C2DE	31.0(230)	33.4(241)	Ref		34.7(250)	Ref	
ABC1	40.8(713)	31.6(553)	0.86(0.68-1.09)	.21	27.6(482)	0.69(0.55-0.86)	.001
**Age group**							
11-15 years	22.1(294)	44.2(588)	Ref		33.7(448)	Ref	
16-18 years	57.0(649)	18.1(206)	0.20(0.16-0.25)	<.001	24.9(284)	0.35(0.28-0.43)	<.001
**Vaping status**							
Never [a]	32.7(682)	36.0(750)	Ref		31.3(653)	Ref	
Ever	64.2(174)	13.7(37)	0.46(0.29-0.70)	<.001	22.1(60)	0.71(0.49-1.02)	.06
Current	77.0(87)	6.2(7)	0.19(0.08-0.46)[b]	<.001	16.8(19)	0.57(0.31-1.04) [b]	.06
**Smoking status**							
Never	32.2(653)	36.0(728)	Ref		31.8(643)	Ref	
Ever	59.5(191)	17.1(55)	0.58(0.40-0.84)	.004	23.4(75)	0.63(0.44-0.88)	.007
Current	79.8(99)	8.9(11)	0.34(0.16-0.69)[b]	.003	11.3(14)	0.27(0.14-0.51) [b]	<.001

[a] Includes respondents who had never heard of e-cigarettes.
[b] caution, estimates likely unreliable due to low sample sizes.
Reference category is 'interest in trying' any of the brands.
Analyses were adjusted for sex, age group, socioeconomic status, vaping status and smoking status.
All data are unweighted

© 2023 Taylor E et al. *JAMA Network Open.*

eTable 6. Interactions Between Packaging Condition and Vaping and Smoking Status, 2021 ASH Smokefree GB Youth Survey (n=2469)

		Packaging condition					
	Branded 'control' (REF)	Green Standardised			White Standardised		
	%(n)	%(n)	AOR(95% CI)	p	%(n)	AOR(95% CI)	p
**Model 1: No interest by interaction between vaping status and condition**							
Vaping status							
Never [a]	32.6(224)	39.3(275)	1.34(1.07-1.69)	.01	36.0(251)	1.16(0.92-1.45)	.22
Ever	13.3(13)	16.9(15)	1.61(0.71-3.69)[b]	.26	10.7(9)	0.90(0.36-2.27)[b]	.82
Current	2.4(1)	7.4(2)	3.03(0.26-36.01)[b]	.38	9.1(4)	3.83(0.4-36.52)[b]	.24
**Model 2: No interest by interaction between smoking status and condition**							
Smoking status							
Never	32.6(216)	40.1(271)	1.38(1.10-1.75)	.006	35.1(241)	1.12(0.89-1.42)	.34
Ever	15.7(19)	16.2(17)	1.18(0.56-2.49) [b]	.66	20.0(19)	1.47(0.71-3.06)[b]	.30
Current	6.7(3)	11.8(4)	1.60(0.32-8.07) [b]	.57	8.9(4)	1.88(0.38-9.39)[b]	.44

[a] Includes respondents who had never heard of e-cigarettes.
[b] caution, estimates likely unreliable due to small sample sizes
Reference category is 'other', including selecting any of the products and don't know.
Analyses were adjusted for sex, age group, socioeconomic status, vaping status, and smoking status.
All data are unweighted

© 2023 Taylor E et al. *JAMA Network Open.*

eFigure 1. No Interest in Trying Vaping Product by Vaping Status, ASH Youth (n=2469)

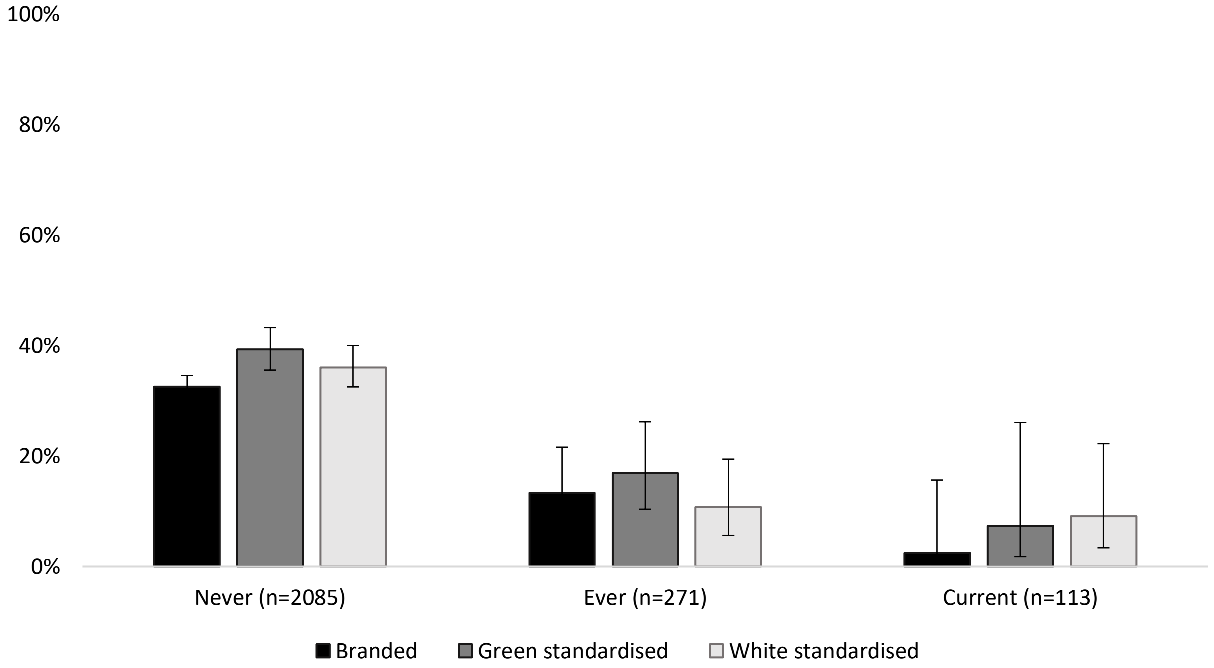

© 2023 Taylor E et al. *JAMA Network Open.*

eFigure 2. No Interest in Trying Vaping Product by Smoking Status, ASH Youth (n=2469)

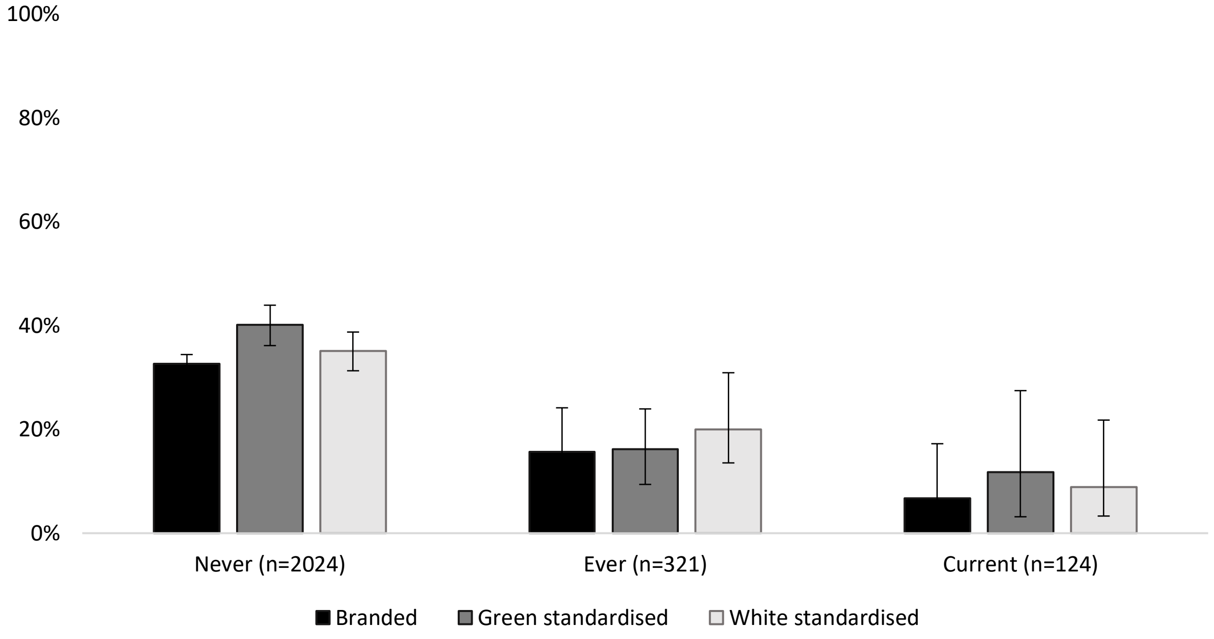

© 2023 Taylor E et al. *JAMA Network Open.*

**eTable 7.** Multinomial Associations Between Reporting No Interest or Don't Know and Packaging Condition, 2021 ASH Smokefree GB Adult Survey (n=12046)							
	Selected a product (ref)	No interest in trying any product shown			Don't know		
	%(n)	%(n)	AOR(95%CI)	p	%(n)	AOR(95%CI)	p
**Total**	7.9(947)	87.7(10563)			4.4(536)		
**Pack condition**							
Branded 'Control'	8.3(331)	88.1(3526)	Ref		3.6(143)	Ref	
Green Standardised	8.0(324)	86.8(3505)	1.02(0.84-1.23)	.86	5.2(211)	1.58(1.21-2.07)	.001
White Standardised	7.3(292)	88.2(3532)	1.25(1.03-1.52)	.02	4.5(182)	1.59(1.21-2.10)	.001
**Sex**							
Male	8.9(503)	86.7(4882)	Ref		4.4(249)	Ref	
Female	6.9(444)	88.6(5681)	1.19(1.02-1.39)	.03	4.5(287)	1.33(1.07-1.66)	.01
**Socioeconomic status**							
C2DE	7.9(396)	86.4(4320)	Ref		5.7(286)	Ref	
ABC1	7.8(551)	88.6(6243)	0.99(0.84-1.16)	.87	3.5(250)	0.74(0.59-0.93)	.009
**Age**							
18-29	15.0(295)	81.2(1598)	Ref		3.8(75)	Ref	
30-39	12.6(241)	82.6(1577)	1.89(1.51-2.37)	<.001	4.8(92)	1.63(1.14-2.34)	.007
40-49	9.6(196)	84.8(1729)	2.43(1.92-3.08)	<.001	5.6(115)	2.44(1.71-3.47)	<.001
50-59	5.6(102)	89.8(1633)	4.22(3.19-5.58)	<.001	4.6(83)	3.23(2.17-4.81)	<.001
≥60	2.6(113)	93.4(4026)	8.39(6.44-10.96)	<.001	4.0(171)	5.96(4.13-8.59)	<.001
**Vaping status**							
Never [a]	2.3(223)	95.5(9156)	Ref		2.2(208)	Ref	
Ever	21.1(228)	68.4(740)	0.23(0.18-0.29)	<.001	10.5(114)	0.55(0.39-0.78)	.001
Current	36.0(496)	48.4(667)	0.06(0.05-0.08)	<.001	15.5(214)	0.42(0.32-0.57)	<.001
**Smoking status**							
Never	2.5(160)	96.0(6139)	Ref		1.5(96)	Ref	
Former	9.2(387)	86.3(3616)	0.47(0.37-0.61)	<.001	4.5(188)	0.92(0.64-1.32)	.64
Current	27.0(400)	55.3(808)	0.17(0.13-0.22)	<.001	17.3(252)	1.39(0.96-2.01)	.08

[a] Includes respondents who had never heard of e-cigarettes.
Reference category is 'other' including interest in trying any of the brands and don't know.
Analyses were adjusted for sex, age group, socioeconomic status, vaping status, and smoking status.
All data are unweighted

© 2023 Taylor E et al. *JAMA Network Open.*

eTable 8. Interactions Between Packaging Condition and Vaping and Smoking Status, 2021 ASH Smokefree GB Adult Survey (n=12046)

	Branded 'control' (REF)	Green Standardised			White Standardised		
	%(n)	%(n)	AOR(95% CI)	p	%(n)	AOR(95% CI)	p
Model 1: No interest in trying by interaction between vaping status and condition							
**Vaping status**							
Never [a]	95.7(3061)	95.0(3059)	0.85(0.67-1.07)	.17	95.8(3036)	0.99(0.77-1.26)	.91
Ever	68.4(234)	62.7(229)	0.75(0.54-1.05)	.09	73.9(277)	1.39(0.98-1.96)	.06
Current	50.1(231)	47.7(217)	0.94(0.71-1.24)	.67	47.5(219)	0.93(0.71-1.23)	.61
Model 2: No interest in trying by interaction between smoking status and condition							
**Smoking status**							
Never	96.0(2041)	95.5(2039)	0.90(0.67-1.22)	.51	96.5(2059)	1.19(0.86-1.65)	.29
Former	87.2(1210)	85.7(1208)	0.87(0.68-1.11)	.26	85.9(1198)	0.93(0.73-1.19)	.55
Current	56.7(275)	52.0(258)	0.79(0.59-1.04)	.10	57.4(257)	1.11(0.83-1.48)	.48

[a] Includes respondents who had never heard of e-cigarettes.
Reference category is 'other' including interest in trying any of the brands and don't know.
Analyses were adjusted for sex, age group, socioeconomic status, vaping status, and smoking status.
All data are unweighted.

© 2023 Taylor E et al. *JAMA Network Open.*

eFigure 3. No Interest in Trying Vaping Product by Vaping Status, ASH Adult (n=12046)

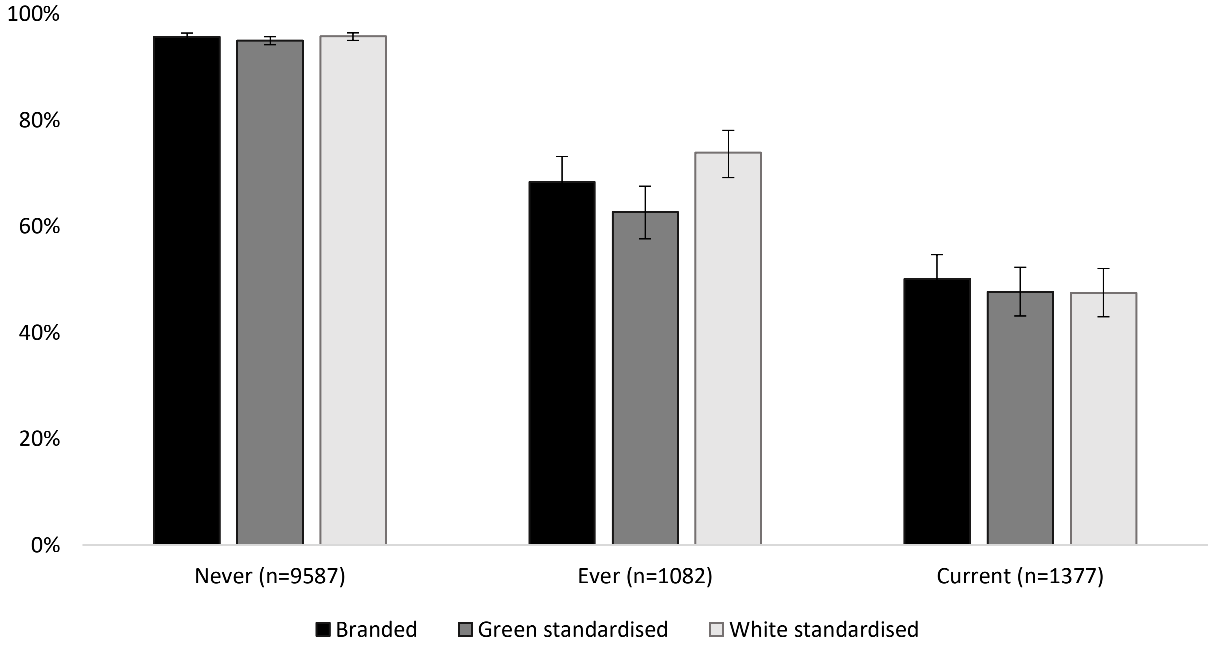

© 2023 Taylor E et al. *JAMA Network Open.*

eFigure 4. No Interest in Trying Vaping Product by Smoking Status, ASH Adult (n=12046)

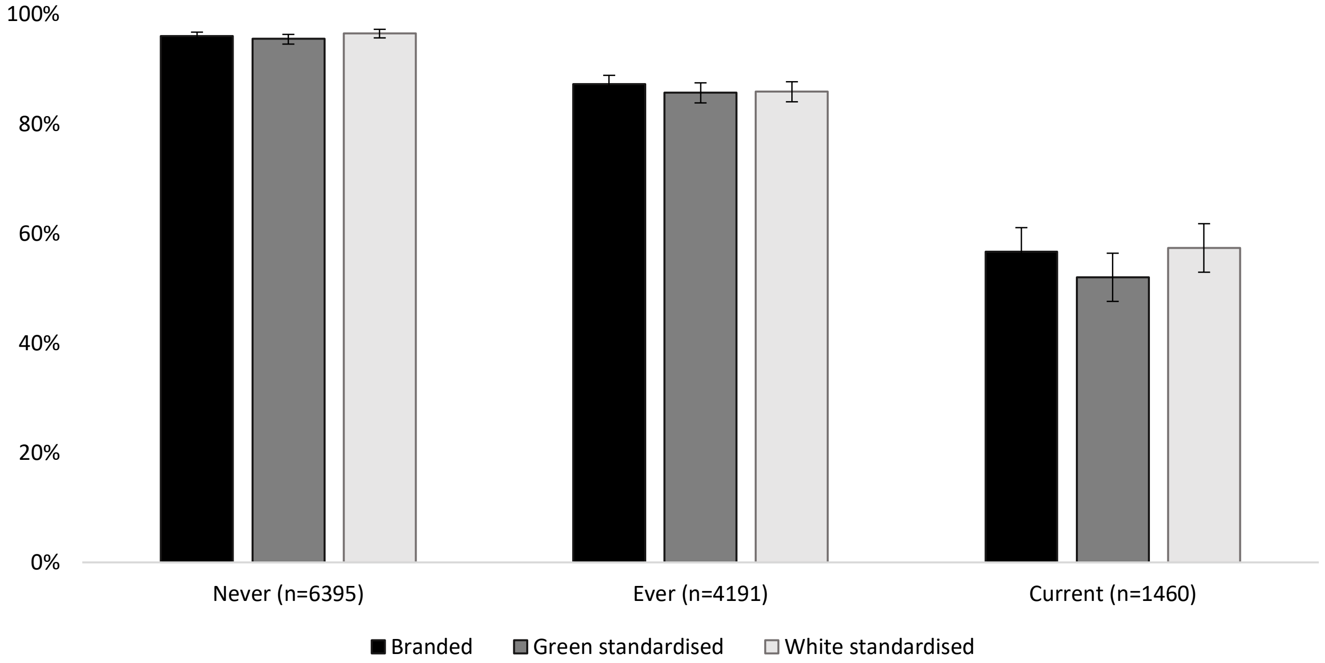

© 2023 Taylor E et al. *JAMA Network Open.*
