# Supplementary material for: Association of Fully Branded and Standardized e-Cigarette Packaging With Interest in Trying Products Among Youths and Adults in Great Britain
Source: JAMA Netw Open. 2023 Mar 14;6(3):e231799. doi: 10.1001/jamanetworkopen.2023.1799 (PMC10015302; doi:10.1001/jamanetworkopen.2023.1799)
Supplement: Supplement 2. — Data Sharing Statement [file jamanetwopen-e231799-s002.pdf]

Data Sharing Statement

Taylor. Association of Fully Branded and Standardized e-Cigarette Packaging With Interest in Trying Products Among Youths and Adults in Great Britain. *JAMA Netw Open*. Published March 14, 2023. doi:10.1001/jamanetworkopen.2023.1799

Data
Data available: Yes
Data types: Deidentified participant data
How to access data: Available on reasonable request to eve.v.taylor@kcl.ac.uk
When available: With publication

Supporting Documents
Document types: None

Additional Information
Who can access the data: researchers whose proposed use of the data has been approved
Types of analyses: for a pre-specified purpose
Mechanisms of data availability: after approval of a proposal
